# Influence of heat stress and nighttime recovery on ovarian follicle population, oocyte yield, and in vitro embryo production in Holstein heifers

**DOI:** 10.5194/aab-69-301-2026

**Published:** 2026-05-19

**Authors:** Muhammed Furkan Ciftci, Ömer Faruk Yesilkaya, Maide Gölbası, Ayse Sarı, Sakine Ülküm Cizmeci, Dursun Ali Dinc

**Affiliations:** 1 Department of Obstetrics and Gynecology, Faculty of Veterinary Medicine, Selcuk University, Konya, Türkiye; 2 Department of Obstetrics and Gynecology, Faculty of Veterinary Medicine, Muğla Sıtkı Kocman University, Muğla, Türkiye; 3 Department of Reproduction and Artificial Insemination, Faculty of Veterinary Medicine, Necmettin Erbakan University, Konya, Türkiye

## Abstract

The objective of this study was to evaluate the effects of heat stress and nighttime temperature drop on oocyte yield and in vitro embryo production (IVEP) success. The study was conducted on Holstein heifers within the same herd, under similar management and feeding conditions. Each session of ovum pick-up (OPU) was classified into continuous heat stress (CHS; THImax ≥72 and THImean ≥68 ), high daytime temperature–humidity index (THI) with nighttime recovery (HDT-NR; THImax ≥72 and THImean <68 ), and thermoneutral (TN; THImax <72 and THImean <68 ) groups based on environmental THI values. The TN group was found to have a greater number of small- to medium-sized follicles and a higher total number of follicles in comparison to the CHS group ( p<0.05 ). In terms of oocyte yield, the numbers of grade-A, total, and viable oocytes were found to be higher in the TN and HDT-NR groups than in the CHS group ( p<0.05 ). The assessment of the IVEP revealed that the mean number of blastocysts per OPU was lower in the CHS group ( p<0.05 ). Consequently, even when the daytime maximum THI was high, nighttime cooling, reflected by a lower mean THI, appeared to preserve OPU and IVEP performance without adverse effects. Therefore, during the summer season, when the likelihood of heat stress is increased, considering nighttime recovery while planning and conducting in vitro embryo production processes may be an important criterion for improving production success.

## 1 Introduction

In vitro embryo production is an advanced biotechnological approach developed to accelerate genetic progress in cattle breeding, facilitate the dissemination of superior genotypes, and optimize reproductive efficiency (Baruselli et al., 2018). The success of this technology largely depends on the number and quality of oocytes retrieved by the ovum pick-up (OPU) procedure (Watanabe et al., 2018). OPU and in vitro embryo production (IVEP) procedures enable the collection of a substantial number of oocytes at brief intervals without exerting any direct influence on the physiology of donor animals. This facilitates the production of a greater number of embryos from animals that possess high genetic value (Karasahin et al., 2021; Ferré et al., 2023). However, environmental factors, particularly high ambient temperature, play a decisive role in this process, influencing oocyte quality and embryo development (Berling et al., 2022; Khan et al., 2023).

Heat stress is a type of environmental stress that occurs when the thermoneutral range of cattle is exceeded. It is usually defined by the temperature–humidity index (THI) (Dash et al., 2016). It has been documented that elevated THI levels, exceeding 72, are associated with a range of physiological responses in livestock, including an increase in body temperature, a decrease in feed intake, and irregularities in endocrine function (Miętkiewska et al., 2022). This condition disrupts the activity of the hypothalamic–pituitary–ovarian axis, negatively affecting gonadotropin and steroid hormone secretion. These disruptions may negatively affect follicular development, oocyte maturation, and embryo development (Penev et al., 2021). Increased oxidative stress and decreased blood flow in the follicular environment limit the metabolic activity and mitochondrial function of the oocyte, thereby reducing its developmental capacity (Roth, 2017).

It has been reported that, under conditions of heat stress, the maturation of the oocyte, both nuclear and cytoplasmic, is impaired; the ability to fertilize is reduced; and arrests occur in the early stages of embryonic development (Gómez-Guzmán et al., 2024). Particularly during periods of persistently high ambient temperatures, increased apoptosis in granulosa cells and decreased mitochondrial energy production limit the developmental capacity of the oocyte (Held-Hoelker et al., 2025). However, during certain periods, the decline in ambient temperature at night, despite high daytime temperatures, may allow for a partial balance in the physiological parameters of animals. This phenomenon is known as nighttime recovery and is considered to be a factor that allows the organism to partially tolerate the heat load (Bertens et al., 2024; Gómez-Guzmán et al., 2024; López-Gatius, 2025).

There are some differences regarding the classification of heat stress. The THI threshold values vary across different studies, leading to ambiguities in defining stressful and non-stressful periods (Brügemann et al., 2012). It has been reported that, despite high daytime temperatures during certain periods, the temperature drops at night, making the condition physiologically tolerable (Bertens et al., 2024). Therefore, when assessing heat stress, it is important to consider not only maximum temperature values but also daily average temperatures. This is because both daytime thermal load and nighttime temperature variations must be taken into account. This approach allows for a more accurate assessment of the true implications of heat stress on reproductive physiology and the potential effects of nighttime temperature drops on oocyte quality and embryo development (Tian et al., 2021; Cholakkal, 2025). However, the impact of THI threshold values and nighttime temperature drops on OPU and IVEP results is not fully understood in the literature.

The study evaluated the effects of heat stress and nighttime temperature drops on the success of OPU and IVEP applications in cattle under different environmental conditions defined according to the THI. The objective of this study is to ascertain the impact of heat stress and nighttime recovery under varying thermal conditions on oocyte yield and in vitro embryo production.

## 2 Materials and methods

### 2.1 Animals and location

In this study, Holstein heifers aged 12–24 months with good general health and no pathologies detected in terms of clinical examination findings were used as material. The animals were fed ad libitum; the ration was balanced to include roughage sources such as hay, alfalfa silage, corn silage, and alfalfa along with concentrate feed and was also supplemented with appropriate vitamin and mineral supplements. At the farm, animals were kept in free-stall shelters and had constant access to feed and water. The shelters had adequate ventilation, and there were no additional environmental or managerial stressors, such as overcrowding, malnutrition, or poor ventilation.

The study was conducted over a 12-month period and was designed to encompass the effects of temperature and humidity conditions observed in different seasons of the year. The present study was conducted at a dairy cattle farm in the Konya province of Türkiye (37°34^′^17^′′^ N, 32°47^′^04^′′^ E). Konya is located at an altitude of approximately 1016 m above sea level. The region exhibits a semi-arid continental climate, characterized by hot, dry summers and cold winters with relatively low precipitation.

### 2.2 Meteorological data and THI calculation

The daily meteorological data (air temperature and relative humidity) utilized in the present study were obtained from the NASA POWER (https://power.larc.nasa.gov, last access: 25 December 2025) reanalysis database. The meteorological parameters in the NASA POWER data are derived from the Modern-Era Retrospective Analysis for Research and Applications, Version 2 (MERRA-2) atmospheric re-analysis products generated by NASA GMAO. These parameters represent the average values of the respective grid cell (Gelaro et al., 2017). A comparison of the data with ground observation data from different geographical regions has shown that the data yield accurate and reliable results (Rockett et al., 2023; Halimi et al., 2023).

The THI values were calculated using ambient temperature and relative humidity data from the days on which the OPU procedure was performed. The THI is calculated using the following formula, based on air temperature and relative humidity values:

- THI =0.8×T+RH/100×(T-14.4)+46.4 ( T : average or maximum daily air temperature (°C); RH: percentage of relative humidity (%)). This formula is one of the most frequently employed techniques for determining the environmental heat load in dairy cattle (Armstrong, 1994; Mader et al., 2006).

### 2.3 Oocyte collection

During the transvaginal oocyte aspiration procedure, a collection unit connected to a combined catheter aspiration system (bovine OPU aspiration pump, 230 V, Minitube) was used. The determination of follicle diameter and ultrasonographic applications throughout the aspiration process were performed using a real-time ultrasound system (Esaote MyLab TwiceVet, 5001) with a microconvex probe (SC3123 VET, Esaote) operating at a range of 4.0–9.0 MHz. Prior to OPU, follicular structures observed on the ovary were categorized and recorded according to their diameters. During the aspiration process, all follicles with a diameter greater than 2 mm in the ovary were aspirated using a 20 G catheter needle.

### 2.4 THI classification and experimental design

OPU sessions were conducted on Holstein heifers from the same herd, under conditions of similarity with regard to management and feeding. The classification of each OPU session was determined by the prevailing environmental THI conditions at the time. A total of 119 OPU sessions were conducted during the study period. In order to evaluate the effect of heat stress on oocyte retrieval results, all OPU sessions were divided into three different groups based on their THI values. THImax was defined as the THI value calculated based on the highest temperature recorded on an OPU day, while THImean was defined as the THI value calculated using the average temperature of the same day.

- *Continuous heat stress (CHS, n* = *36).* This group included sessions with THImax values ≥72 and THImean values ≥68 , representing OPU sessions performed under continuous heat stress.
- *High daytime THI with nighttime recovery (HDT-NR, n* = *41).* This group included sessions with THImax values ≥72 and THImean values <68 . This situation represents conditions in which short-term heat stress occurs during the daytime, but thermal recovery takes place as temperatures drop at night.
- *Thermoneutral (TN, n* = *42).* This group includes sessions with THImax <72 and THImean <68 . These conditions refer to environmental situations where heat stress is absent.

The determination of THI threshold values was informed by extant literature, with particular reference to studies defining thermal comfort and stress ranges in cattle. It is widely accepted that when the THI value exceeds 72, the thermal comfort limit of animals is surpassed, thereby inducing heat stress conditions. Moreover, when the THI falls below 68, the ambient temperature is at a level at which animals can physiologically re-establish equilibrium and achieve thermal recovery (Polsky and Von Keyserlingk, 2017; Adhikari et al., 2022). Therefore, in this study, conditions with THI ≥72 were considered to be heat stress, and conditions with THI <68 were considered to be the thermal recovery period. In this classification, a THImean value below 68 is considered to be an indicator reflecting the decrease in heat load and thermal recovery that occurs as the temperature drops during nighttime hours. Each OPU session was regarded to be an independent experimental unit. Prior to the OPU sessions, no hormonal treatment was administered, and oocyte retrieval was performed on a random day of the estrous cycle.

### 2.5 Oocyte classification and IVEP

The morphological classification of the cumulus–oocyte complexes (COCs) was performed under a stereomicroscope. The oocytes were evaluated based on the compactness of the cumulus cells, the integrity of the cumulus layers, and the homogeneity of the cytoplasm. In accordance with these criteria, COCs were classified into four quality levels: very good (A), good (B), medium (C), and poor (D). The IVEP process exclusively incorporated oocytes originating from quality groups A, B, and C. COCs with homogeneous cytoplasm, regular morphology, and at least one layer of compact cumulus cell structure were considered to be viable (Petyim et al., 2003; Hayden et al., 2022).

The production of in vitro embryos was accomplished through the utilization of commercial culture media (IVF Bioscience; BO-OPU, BO-IVM, BO-IVF, BO-IVC, BO-Wash, BO-Oil, BO-SemenPrep, Cornwall, United Kingdom). Subsequent to the process of quality selection, the COCs were subjected to a wash in a BO-Wash environment and transferred to BO-IVM medium for maturation. The in vitro maturation procedure was conducted at a temperature of 38.5 °C and in a 5.5 % ${\mathrm{CO}}_{2}$ atmosphere for a duration of 20–22 h. Subsequent to maturation, oocytes were transferred to BO-IVF medium for fertilization; semen from the same bull was prepared in BO-SemenPrep medium and used for insemination. The process of fertilization was conducted for a duration of 20 h under conditions of 38.5 °C and 5 % ${\mathrm{CO}}_{2}$ (Alkan et al., 2023).

Subsequent to the process of fertilization, oocytes were separated from cumulus cells by means of vortexing. Putative zygotes were transferred to BO-IVC medium for the purpose of culture. Subsequently, the putative zygotes were incubated for 7 d at 38.5 °C in a droplet culture system covered with BO-Oil in an atmosphere containing 6 % ${\mathrm{CO}}_{2}$ and 6 % O_2_. The development stages of the embryos and the subsequent quality grading were performed in accordance with the standards set out by the International Embryo Technology Society (IETS) (Bó and Mapletoft, 2013; Alkan et al., 2025).

### 2.6 Statistical analysis

The statistical analysis of the data was conducted utilizing SPSS 27.0 software (IBM SPSS Statistics for Windows, Version 27.0; Armonk, NY, USA: IBM Corp.). Prior to analysis, the data were evaluated in terms of basic statistical assumptions. The Kolmogorov–Smirnov test was applied in order to verify the normality preconditions of the variables. Descriptive statistics are presented as mean ± standard error (SE). One-way analysis of variance (ANOVA) was employed to compare embryo production parameters between groups; if a significant difference was identified, a post hoc Tukey HSD test was applied to ascertain which groups were responsible for the discrepancy. Categorical data (oocyte recovery rate, cleavage rate, blastocyst rate) were analyzed using the Chi-square test; when the overall test was found to be significant, the Z test was used to determine differences between groups. The statistical significance level was considered to be p<0.05 .

## 3 Results

**Figure 1:**
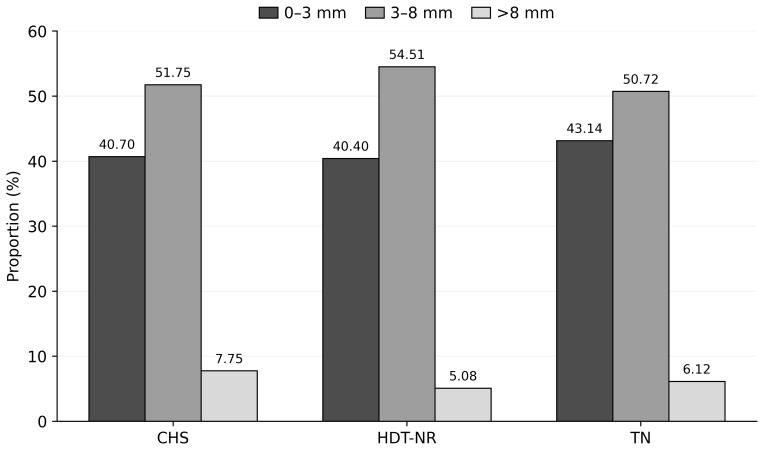
Distribution of follicles within groups according to diameter categories (%).

As demonstrated in Table 1, the mean diameters of follicles are presented for the groups that have been categorized according to their degree of heat stress during the OPU sessions. The findings indicate that the number of small- and medium-sized follicles (0–3 and 3–8 mm), in addition to the total number of follicles, exhibited higher values in sessions devoid of heat stress when compared to sessions involving continuous stress ( p<0.05 ). However, no statistically significant difference was found between the groups in terms of the number of large follicles. The percentage distribution of follicles according to their diameters within the groups is demonstrated in Fig. 1. There was no statistically significant difference between the groups in terms of the percentage distribution of follicle population according to diameters.

**Table 1:** The average number of follicles in the ovary, categorized by diameter, during oocyte retrieval among the groups (mean ± standard error).

Follicle	CHS	HDT-NR	TN
0–3 mm	$8.69\pm 0.81^{a}$	$10.27\pm 0.83^{a,b}$	$12.07\pm 0.98^{b}$
3–8 mm	$11.05\pm 0.86^{a}$	$13.85\pm 1.11^{a,b}$	$14.19\pm 1.85^{b}$
8 mm <	1.61±0.32	1.29±0.21	1.71±0.23
Total	$21.36\pm 1.34^{a}$	$25.41\pm 1.24^{a,b}$	$27.97\pm 2.49^{b}$

^a,b^ The statistical difference within rows ( p<0.05 ).

Data on oocyte yield and the in vitro embryo production process among groups according to stress level are presented in Table 2. The number of grade-A oocytes collected was found to be lower in the continuous-stress group compared to in the thermoneutral group ( p<0.05 ). Although no statistically significant difference was found between the groups in terms of grade-B oocyte count, grade-C and grade-D oocyte counts were found to be higher in the nighttime recovery group compared to the other groups ( p<0.05 ). The findings of the study demonstrated that the total and viable oocyte numbers obtained after OPU sessions were statistically higher in the thermoneutral and nighttime recovery groups. Furthermore, it was determined that the oocyte recovery rate was higher in the nighttime recovery group in comparison to in the other groups ( p<0.05 ).

**Table 2:** Oocyte and embryo development parameters according to THI classification (mean ± standard error).

Variables	CHS	HDT-NR	TN
Grade-A oocytes/OPU	$2.05\pm 0.32^{a}$	$2.51\pm 0.29^{a,b}$	$3.54\pm 0.38^{b}$
Grade-B oocytes/OPU	1.63±0.31	1.75±0.24	2.23±0.35
Grade-C oocytes/OPU	$1.83\pm 0.32^{a}$	$3.04\pm 0.35^{b}$	$2.04\pm 0.29^{a}$
Grade-D oocytes/OPU	$1.36\pm 0.30^{a}$	$3.12\pm 0.39^{b}$	$2.19\pm 0.41^{c}$
Total oocytes/OPU	$6.91\pm 0.91^{a}$	$10.43\pm 0.87^{b}$	$10.07\pm 1.01^{b}$
Oocytes recovery rate (%)	32.37^a^	41.07^b^	36.62^a^
Viable oocytes/OPU	$5.55\pm 0.69^{a}$	$7.22\pm 0.64^{b}$	$7.91\pm 0.68^{b}$
Cleaved oocytes/OPU	$3.88\pm 0.52^{a}$	$6.19\pm 0.58^{b}$	$6.64\pm 0.63^{b}$
Cleavage rate (%)	56.22^a^	59.36^a^	65.96^b^
Blastocysts/OPU	$0.92\pm 0.17^{a}$	$1.59\pm 0.24^{b}$	$1.79\pm 0.23^{b}$
Blastocyst rate (%)	16.50^a^	21.96^b^	22.59^b^

^a−c^ The statistical difference within rows ( p<0.05 ).

In oocyte development assessments performed during in vitro embryo production, the number of cleaved oocytes was found to be higher in the TN and HDT-NR groups compared to in the CHS group ( p<0.05 ). Furthermore, the cleavage rate was observed to be statistically higher in the thermoneutral group than in the other groups. The number of blastocysts obtained per OPU session was found to be lower in the continuous-heat-stress group compared to in the other groups ( p<0.05 ). The blastocyst rate was found to be higher in the thermoneutral and nighttime recovery groups ( p<0.05 ).

## 4 Discussion

The present study investigated the effects of heat stress and nighttime recovery on bovine oocyte yield and in vitro embryo production success. The findings indicate that heat stress exerts a significant adverse effect on follicle development, oocyte quality, and embryo development and that nighttime recovery can partially compensate for these adverse effects.

The observation of a reduced number of small- and medium-sized follicles in heifers subjected to continuous heat stress suggests a general suppression of folliculogenesis by high ambient temperatures. It is known that heat stress exerts a disruptive effect on the hypothalamo–pituitary–gonadal axis, consequently affecting gonadotropin secretion (Wrzecińska et al., 2021; Penev et al., 2021). This hormonal imbalance has been stated to reduce the formation of new antral follicles and increase follicular atresia, resulting in a decrease in follicle count (Roth, 2017). Indeed, it has been reported that, when THI levels rise above 72, body temperature increases; feed intake decreases; endocrine functions are impaired; and, consequently, a significant decrease in fertility can be observed (Dash et al., 2016; Miętkiewska et al., 2022). In this study, the lower number of small ( <3 mm) and medium (3–8 mm) diameter follicles in the group under continuous heat stress compared to the thermoneutral group reflects a general suppression of follicular development due to decreased gonadotropin support and follicular atresia. Conversely, the observation that the number of large follicles ( >8 mm) remained constant between the groups indicates that the development of the dominant follicle might not have been fully suppressed, even under conditions of heat stress. Additionally, the lack of difference in large follicles may be attributed to the limited number of dominant follicles, which inherently reduces variability, whereas small and medium follicles represent a larger and more dynamic pool that is more susceptible to environmental influences (Hansen, 2019). The absence of a difference in the percentage distribution of follicle sizes suggests that heat stress exerts an effect on the entire follicle population as opposed to exerting a selective influence on a particular follicle cohort (De Rensis et al., 2021).

In the context of oocyte quality, it has been observed that elevated temperatures result in a decline in the number of grade-A oocytes. This finding is consistent with the existing body of literature, which demonstrates that heat stress can disrupt oocyte morphology and maturation. Kanzawa et al. (2025) reported that total and quality oocyte numbers decreased and that blastocyst development was negatively affected under hot conditions. Furthermore, it has been stated that thermal stress increases intraoocyte oxidative stress and DNA damage, leading to morphological deterioration; therefore, a greater number of low-quality or damaged oocytes emerge (Alves et al., 2014). Studies have shown that heat stress increases the rate of poor-quality oocytes by disrupting COC compactness and cytoplasmic homogeneity (Lee et al., 2023; Baruselli et al., 2023; Mzedawee et al., 2024). In this study, the higher number of C- and D-grade oocytes observed in the nighttime recovery group compared to in the other groups may be attributed to an overall increase in oocyte yield under lower nighttime temperatures, whereby the absolute number of low-quality oocytes also increased proportionally. This finding suggests that, although follicular activity and oocyte retrieval were partially improved, nighttime recovery was insufficient to fully preserve oocyte quality.

The elevated total number of oocytes and viable oocytes observed in the thermoneutral and nighttime recovery groups in comparison to the continuous-heat-stress group reveals that a chronic heat load has a detrimental effect on oocyte yield. Several studies have reported that heat stress increases the proportion of follicles without oocytes in the ovary, consequently reducing the number of oocytes obtained through OPU (Torres-Júnior et al., 2008; Roth, 2017). However, the total and viable oocyte numbers were found to be higher in the nighttime recovery group compared to the continuous-stress group. This finding suggests that the temperature drop during nighttime hours may have a partial protective effect on OPU efficiency by limiting the heat load accumulated during the day. The main aim of cooling treatments is to shorten the period of hyperthermia. Studies emphasize that reducing the total amount of time that body and vaginal temperatures remain above critical thresholds is crucial for reproductive performance (Wolfenson and Roth, 2019; Roth, 2020). The thermal relaxation that occurs during the nighttime hours may have enabled a partial normalization of ovarian function, resulting in an increase in the number of follicles containing healthy oocytes (López-Gatius, 2025). The highest recovery rate was observed in the nighttime recovery group, suggesting that the partial reduction in thermal load during nighttime hours may have supported oocyte aspiration efficiency to some extent. The fact that it was found to be higher than in the thermoneutral group may be due to factors that cannot be directly controlled, such as individual physiological responses or tolerance levels (Spiers et al., 2018; Hidaka et al., 2018).

This study also observed the negative effects of heat stress on in vitro embryo development. In the continuous-heat-stress group, the number of cleaved oocytes was lower than in the other groups. This finding may be attributed primarily to the reduced total oocyte yield at OPU and, in parallel, to a lower developmental competence of the oocytes selected for IVEP. In addition, the higher cleavage rate observed in the thermoneutral group suggests both an increase in the total number of cleaved oocytes and a greater suppression of early cleavage capacity under heat stress. Indeed, it has been reported that high temperatures can cause developmental blockages and delays in the early stages of oocyte division (Payton et al., 2004; Pavani et al., 2015). Similarly, it has been reported that embryos obtained from oocytes collected during the warm season undergo developmental arrest more frequently and experience increased embryonic loss during the pre-implantation period (Sakatani, 2017). Gendelman et al. (2010) also emphasized that the proportion of embryos that reach the blastocyst stage decreases when oocytes are collected during the summer months. In the presented study, the number of blastocysts per OPU was found to be lower in the continuous-heat-stress group compared to in other groups. Moreover, the observation of a higher blastocyst rate in the thermoneutral and nighttime recovery groups suggests that heat stress may not only reduce the number of blastocysts but also negatively affect the efficiency of blastocyst development in cleaved embryos. Previous studies have reported that heat stress reduces blastocyst formation, whereas appropriate cooling strategies and lower nighttime temperatures may mitigate this adverse effect (Wolfenson and Roth, 2019; López-Gatius, 2025). Furthermore, Kadokawa et al. (2012) reported that effective cooling during oocyte maturation and early embryonic development can reduce the adverse effects of heat stress and increase pregnancy rates. The study found that the blastocyst yield was higher in the group that experienced nighttime recovery than in the group exposed to continuous heat stress. This finding indicates that a partial reduction in heat load during nighttime hours may enhance IVEP success by supporting the developmental process from oocyte to blastocyst.

## 5 Conclusion

Consequently, OPU and IVEP performance was less negatively affected under conditions where a decrease in nighttime temperature was observed compared to conditions with continuous heat load. Although high daytime temperatures might suggest stress, performance indicators did not deteriorate significantly during sessions characterized by a natural decline in nighttime temperature. Even when the maximum temperature was the same, the indicators of embryo production were better in the group that showed nighttime recovery than in the group that experienced continuous heat stress. Therefore, it is recommended that not only daytime maximum temperatures, but also the degree of nighttime recovery and daily average temperatures be routinely considered in heat stress assessment.

## Data Availability

The datasets used and analyzed during the current study are available from the corresponding author on reasonable request.
